# Establishing a Pre-COVID-19 Hospital Biopreparedness Initiative

**DOI:** 10.1017/dmp.2021.91

**Published:** 2021-03-25

**Authors:** Saskia Popescu, Rebecca Leach, Krystal Robinson

**Affiliations:** 1 George Mason University, Schar School of Policy and Government, Fairfax, VA, USA; 2 HonorHealth, Scottsdale, AZ, USA

**Keywords:** biodefence, infectious disease transmission, public health surveillance, disease outbreaks, biosurveillance

## Abstract

The severe acute respiratory syndrome coronavirus-2 (SARS-CoV-2)/coronavirus disease 2019 (COVID-19) pandemic has amplified the role of hospitals in infectious disease response and capacity building. In efforts to respond to the growing volume of cases, hospitals have become a microcosm for US pandemic response. The COVID-19 outbreak has highlighted that hospital preparedness for biological events, such as a pandemic, are often inadequate and dependent on leadership investment in biopreparedness. This article discusses the proactive decision, before COVID-19, that a Phoenix-based hospital system made to invest in high-consequence disease (HCD) preparedness. Within these efforts, a gap analysis was performed, which led to creation of an HCD subcommittee and corresponding efforts to address vulnerabilities and opportunities for improvement. From establishing enhanced personal protective equipment (PPE) and infectious disease training for frontline staff, to building an outbreak tracking mechanism for travel alerts within the electronic medical record, the HCD efforts of this hospital system created a stronger foundation to respond to biological events like the COVID-19 pandemic.

The severe acute respiratory syndrome coronavirus-2 (SARS-CoV-2)/coronavirus disease 2019 (COVID-19) pandemic has soared past 145 million cases internationally and been a stark reminder of the serious threat of emerging and novel infectious diseases. In the wake of the pandemic, there will surely be lessons learned regarding issues ranging from supply chain to laboratory testing, and public health resources. For many though, none will be as painfully obvious as that of health-care preparedness and response.

Pandemic preparedness in health care is challenged by many roadblocks. How do we make the case for investing in costly efforts that might never be used? Buying additional personal protective equipment (PPE) and ventures to provide enhanced training for staff are not always a priority for hospital leadership, especially in times of economic strain and soaring health-care costs. A 2018 Office of the Inspector General for the US Department of Health and Human Services report found that, while hospital leaders reported improved preparedness of emerging infectious diseases after the 2014/2016 Ebola outbreak, they reported competing priorities that often limited the ability to invest in continued preparedness.^1^ Biopreparedness efforts are those that seek to identify and address vulnerabilities to a spectrum of biological threats within health-care settings. From natural events such as pandemics to intentional threats such as bioterrorism, biopreparedness efforts focus on enhancing readiness to infectious diseases and an awareness of the serious implications they pose to health-care infrastructure. Making the case for biopreparedness is not easy, but these efforts are built on a foundation of infection prevention and control (IPC) that can have far reaching benefits.^2^

Within this article, we detail the efforts and process of establishing a high-consequence disease (HCD) subcommittee and initiative beginning in 2018 and how this enabled enhanced readiness for the COVID-19 pandemic.

## Laying the Groundwork: A Gap Analysis and Formulation of the HCD Subcommittee

In late 2018, under new leadership within a large medical system in Phoenix, Arizona, the IPC team was able to form a new initiative to address biopreparedness gaps across six hospitals and nearly 100 outpatient facilities. Led by the IPC program director and an infection preventionist with a background in biodefense, efforts were made to identify what biopreparedness would mean for a large hospital system. Once senior administrative support was established, the first phase involved a gap analysis to understand the vulnerabilities and opportunities for improvement. The gap analysis was designed by using Ebola readiness and response requirements from the Centers for Disease Control and Prevention (CDC), as well as the National Emerging Special Pathogens Training and Education Center, as well as personal experiences. The decision was made to use Ebola virus disease as the testing scenario as it was not only the most recent experience staff had with an HCD, but also required the most enhanced measures in terms of response and readiness from PPE to environmental disinfection. All hospitals within this health-care system are classified as frontline facilities within the special pathogens tiered hospital network, meaning that the expectation is that they identify, isolate, and inform potential patients with special pathogens and be prepared to care for them for 12-24 h, and transfer them to an assessment or treatment facility.^3^

The gap analysis evaluated readiness from the moment a patient walked into an emergency department and throughout their care to the point of discharge. It included 44 points within several categories: emergency department, laboratory services, impatient processes, transportation and security, environmental services and waste management, materials management, information technology, human resources, and IPC. Data points included components such as patient admission intake inclusion of travel history, designated PPE donning and doffing areas, training competencies for collection of laboratory specimens, etc. The gap analysis included aspects such as availability of enhanced PPE and responsibility for maintaining kits, but also travel alerts within the electronic medical record system. Because IPC was primarily responsible for Ebola readiness during the 2014/2016 outbreak, it was also important to include components such as infection preventionist training in Ebola PPE, tracking of exposed staff, and if the IPC program had been maintaining an annual assessment and plan for HCD response.

Upon analysis and review, there was considerable opportunities for improvement. The majority of these centered around tacit knowledge and awareness regarding PPE and processes within the emergency department. Gaps included awareness of where one could find the necessary PPE, logs for those entering the room of a patient under investigation for Ebola, knowledge of the communication plan or how to set up a patient room, ability to document relevant travel history or exposure to an HCD in a manner that would alert staff, etc. The gap analysis revealed a deep need for not only PPE training, but also a revision and re-education on processes within the health-care system to respond to patients with an HCD. Last, it also pointed to a need for continued monitoring within the IPC program, of ongoing outbreaks of emerging infectious diseases or special pathogens that might pose a risk for international transmission.

Following the gap analysis, a subcommittee was established to help address each vulnerability and create a unified response across the health-care system. One of the primary goals of this initiative was to establish a written plan that involved all facets of HCD response and could easily be accessed. The goal of the HCD subcommittee and the written plan was to approach biopreparedness, not just for viral hemorrhagic fevers, but also respiratory pathogens, and unknown pathogens, as the World Health Organization has classified “Disease X” as a priority pathogen for research and development.^4^ Preparing the hospitals and staff for a range of biological scenarios was important to ensuring an all-hazards approach that focused on building foundational knowledge and critical thinking during complex, high-stress biological events. The plan included sections on everything from cohorting patients to waste management to PPE kits and laboratory processes for patients under investigation for an emerging pathogen. Last, the plan included a quick guide that consisted of 4 pages that could easily be accessed and guide staff through initial response, including setting up an anteroom barrier for PPE doffing, donning/doffing steps, necessary labs, and cleaning materials.

While the written plan was one aspect of standing up biopreparedness, the next was engaging those critical to enhancing readiness within the hospital system. The HCD subcommittee was established in early 2019 and involved key stakeholders including leadership and representatives from the following departments: laboratory, facilities, environmental services, human resources, occupational health, risk, regulatory, nursing, emergency department medical providers, emergency management, and infection prevention. The HCD subcommittee established a monthly meeting schedule and quarterly goals. Between each meeting, the infection prevention leaders of the HCD team met with key stakeholders from each area involved in the gap analysis to help address vulnerabilities and review their section of the HCD plan. This portion was critical for not only engagement, but also to ensure there was unification between the plan and actual processes.

The HCD subcommittee worked to address several immediate issues within the hospital system, including PPE kits for care of patients with viral hemorrhagic fever, designation of specific rooms within each emergency department that would act as an “HCD room” and could support the building of an emergency anteroom, and establishing classes for frontline staff to educate on HCDs and the PPE processes required for safe care. From January to December 2019, the HCD subcommittee worked to create PPE kits with all the necessary materials to care for patients with viral hemorrhagic fever. Sixty kits were available in each emergency department with a reserve in each hospital supply chain department. Classes were created for frontline staff with the expectation that 90% of emergency department health-care workers attend. These classes were held at all hospital campuses and at 2 urgent care locations, with Continuing Education credits available for those who completed the 90-min course. The course involved 30 min of lecture on what HCDs were, hospital efforts to prepare for them, and various aspects of the HCD plan, such as workflow to don PPE, enter the patient room, doff PPE in the anteroom, and safely leave the space. The second portion of the course focused on donning and doffing PPE, as well as performing tasks to understand the challenges of working in enhanced PPE. The committee was also able to create a new travel screening tool within the electronic medical record that created an alert for any patient who indicated travel to a region with an active outbreak of an emerging infectious disease. Last, each hospital successfully completed a test of emergently building the barriers for an anteroom outside the designed HCD room. The test involved building the barriers in coordination with emergency department leadership, IPC, and facilities.

All of these efforts, including additional endeavors like establishing a waste management contract for hazardous waste and testing of airborne infection isolation rooms (AIIR), were reported to the committee. Moreover, the infection preventionist at each hospital reported on the creation and status of the HCD efforts at several hospital leadership meetings to ensure dissemination of information and awareness of the health-care system efforts. In the midst of ramping up HCD class availability and training though, an outbreak of a novel coronavirus began gaining traction in early 2020.

## Shifting Gears: Harnessing the Power of HCD Efforts for COVID-19 Response

Due to the biopreparedness efforts within the HCD subcommittee, the IPC team had established continued monitoring of international outbreaks and began reporting on the novel coronavirus outbreak to the subcommittee in early January 2020. During this time, a best practice advisory (BPA) alert within the electronic medical record system was created for patients with recent travel to Wuhan City, China, which would alert health-care providers of an ongoing outbreak and consideration for isolation precautions with links to the CDC website on COVID-19. The subcommittee focused on the novel outbreak during a portion of the January meeting and for the February 2020 meeting, had reporting from each hospital on the status of AIIR efficacy and PPE volumes from supply chain. As the first US cases were identified in late January, efforts were ramped up to convert more HCD subcommittee work to focus on COVID-19 rather than the Ebola outbreak in the Democratic Republic of the Congo.

HCD classes continued with a new focus on educating around the COVID-19 outbreak and the isolation requirements for potential patients. Beginning in mid-February 2020, PPE supplies were reported to the HCD subcommittee, and weekly meetings were created to address COVID-19 preparedness. These weekly meetings focused on reporting outbreak status updates and then processes measures for response, which included PPE, surge capacity plans, laboratory needs, and widespread communication and training efforts. Infection preventionists at each campus also presented to hospital leadership on the outbreak, ongoing plans, and the current state of the outbreak. Furthermore, infection preventionists did focused, intensive in-person COVID-19 PPE training for all staff, focusing on those emerging department, intensive care units, designated cohort units, respiratory therapists, environmental services, and lab personnel. This training was provided for day and night shift and included return demonstrations to ensure staff felt comfortable with the PPE requirements. Moreover, infection preventionists worked with staff in the cohort units to ensure rooms were set up accordingly and workflow was optimal for success and avoidance of cross-contamination. A new isolation guidance and signage was created for Enhanced Respiratory Precautions, which included CDC guidance and provided visual cues to ensure the appropriate isolation precautions were not only used but staff entering the room were aware. Last, 24/7 just-in-time N95 respirator fit-testing was provided as PPE supplies fluctuated.

As a result of the existing HCD infrastructure, modifications to the electronic medical record alerts were easier and allowed for more rapid modification to match evolving guidance from the CDC. This process would later be built out more to provide alerts with directions for proper isolation, testing, and notification processes to ensure staff had the information immediately when the patient was identified as high risk through either self-reported travel or exposure. Through pre-existing alerts to the IPC team for measles and pertussis tests ordered, the team was able to have text and email alerts for those patients identified to meet patient under investigation (PUI) criteria for COVID-19 and then later, positive SARS-CoV-2 tests. This rapid alert ensured there were not communication failures for the IPC team, but also comforted staff in knowing that they were supported. Moreover, the algorithms for patient movement and isolation were used from the HCD plan’s existing respiratory pathogen section. As the HCD trainings had included sections on respiratory pathogens that would include PPE beyond facemasks, these lessons were used for staff training and process measures.

In terms of engineering and environmental control aspects, the HCD committee efforts to ensure functioning AIIR, as well as the designation of cohort units for surges of patients with HCDs, were used for COVID-19 response. Due to a heavy focus on the environmental management of HCDs, disinfection products were rapidly assessed to ensure they met claims for Environmental Protection Agency (EPA) Emerging Viral Pathogens efficacy. Last, the focus on how to prepare a room for a PUI or confirmed case, was used from HCD efforts. This involved removing as much unnecessary equipment or materials from the room and ensuring supplies, including PPE, were on carts outside the room as well as clear signage. Furthermore, the use of ultraviolet-C (UV-C) disinfection robots was incorporated into response. While this process was a strategy for the viral hemorrhagic response portion of the plan, the use of the health-care system UV-C disinfection robot fleet was incorporated into disinfection protocols for all PUI and COVID-19 cohorted areas.

Last, the creation and development of the HCD subcommittee and health-care system plan meant that key stakeholders, including hospital leadership, were aware of the various components and aspects of biopreparedness and response. Because both the plan and subcommittee had addressed the wide variety of factors that go into responding to bioevents, less time was spent convincing leadership or stakeholders of their roles. The ability to begin an early response and initiate preparedness and readiness protocols considerably aided in health-care system’s ability to manage the COVID-19 pandemic. Having leadership support for these efforts, especially in the early days of the outbreak, allowed for a more strategic rather than frenzied response. HCD efforts were translated into Hospital Incident Command System initiation as the network and hospitals began to see more cases and an influx of patients.

## Challenges

While the established HCD subcommittee and efforts aided in response to COVID-19, there were several challenges that we have faced, both internal and external, that will be incorporated into future efforts.

The availability of PPE is one that has been felt globally, but nonetheless stressed efforts.^5^ While we began review of assessment of PPE, especially N95 respirator, use early in the pandemic, this was still a stress and strain on resources. From PPE to disinfecting wipes, the challenges of maintaining a steady stream of supplies was not anticipated, but required a rapid response in terms of using federal protocols for re-use and extended use.

As a result of these new PPE use strategies, as well as the changing federal guidance on masks and respirator requirements, there were challenges in ensuring staff awareness and trust in the new processes. While staff had felt more confident in their PPE skills following HCD classes, having to do emergent education during a pandemic on extended use of N95 respirators was a novel situation and one that required more time addressing questioned protocols. Change in PPE practices coupled with the “infodemic” surrounding staff through social media and other news outlets, became a daily focus for IPC efforts.^6^ Transparency and staff trust in processes has become a key piece in infection prevention efforts during COVID-19 and will need to be incorporated into future HCD efforts.^7^ This aspect of COVID-19 response reinforces the importance of crisis communication and science communication, especially for infection preventionists. While education and communication are critical aspects of infection prevention duties, more emphasis should be placed on ensuring there are training and resources to ensure they are successful communicators for both crisis and daily events.

Last, the limitations in testing were an unexpected hurdle. While we had learned several key lessons in coordinating testing and sample collection during a real-world drill involving a symptomatic patient returning from the Democratic Republic of Congo, the testing roadblocks for COVID-19 were taxing. Coordination of testing with public health had been a learning lesson from our 2019 rule-out-Ebola patient, but the widespread demands for SARS-CoV-2 testing from patients and physicians alike were difficult to manage in the early days of limited testing. In efforts to avoid inundating the local health department, we established a testing request process in which medical providers would call IPC first and if the patient met public health criteria for testing, we would initiate conversations with the local public health department. This process was imperfect and taxing on the IPC team but ultimately resolved when third-party, and later in-hospital, testing became available.

## Future Efforts

Despite the extensive work in developing the HCD subcommittee, there is still much work to be done. Incorporation of the lessons learned from COVID-19 will be built into an extension of the HCD plan, which will be COVID-19 specific. Because there was a considerable amount of process and document development for this pandemic, we are in the process of compiling these efforts in a COVID-19 plan, which would fall under the larger HCD plan. Within this, there will be a collection of the documents and guidance created during the pandemic, but also a review of scaling up and descaling efforts, such as universal masking, which will have trigger points to ensure future response has a clear approach to initiating enhanced infection control measures.

As a result of this pandemic, the HCD training will also be designed to focus less on viral hemorrhagic fevers and more on respiratory pathogens, such as COVID-19. The classes will focus more on the nuances to COVID-19 response that have made it challenging but also reflect the majority of questions and hurdles IPC has faced.

While the United States is still battling the COVID-19 pandemic and there will likely be a renewed response for secondary waves, the creation and development of HCD efforts has aided in readiness. As these are not mandated or regulatory required efforts, we hope that our experiences will encourage other hospital and health-care systems to invest in biopreparedness.^8^ Investing in pandemic and HCD preparedness requires a proactive and willing hospital leadership. The decision to purchase additional and infrequently used PPE kits and support personnel training efforts is one that not every leadership team will view as worthwhile, but our experiences have shown how valuable they can be. Situational awareness and ability to rapidly scale-up efforts have made a considerable difference at our hospital system. Our COVID-19 response efforts have built on the work of the HCD subcommittee, which allowed for a less frenzied, more uniformed and calm approach to pandemic response. As IPC played a significant role in HCD response, our role was further highlighted during COVID-19, which reinforced the critical role of infection prevention during biological events. Furthermore, these efforts facilitated the health-care system in continuing to provide adequate PPE to staff without having to make dangerous compromises or practices. Establishing biopreparedness efforts, such as an HCD subcommittee, can help prepare health-care facilities for future biological events and potentially avoid adverse outcomes. Biopreparedness builds on foundational infection prevention, which strengthens hospital efforts to reduce infectious disease transmission, whether it be a health-care-associated infection or an emerging infectious disease.
